# Unusual case of spontaneous hemopneumothorax in a Tunisian pulmonology department: a case report

**DOI:** 10.11604/pamj.2021.38.274.28646

**Published:** 2021-03-17

**Authors:** Rahma Ben Jazia, Jihene Ayachi, Farouk Chatbouri, Ameni Kacem, Amira Faidi, Dhouha Ben Braiek, Anis Maatallah

**Affiliations:** 1 Pulmonology Department, Ibn El Jazzar University Hospital, Kairouan, Tunisia,; 2 Medical Intensive Care Unit, Ibn El Jazzar University Hospital, Kairouan, Tunisia,; 3 Emergency Department, Ibn El Jazzar University Hospital, Kairouan, Tunisia

**Keywords:** Spontaneous hemopneumothorax, thoracic drainage, thoracoscopic surgery, case report

## Abstract

Spontaneous hemopneumothorax is a rare encountered entity in clinical practice. It can be life threatening, so a prompt diagnosis and therapeutic intervention are required. We report a case of a right spontaneous hemopneumothorax in a 31-year-old man, complicated with hemorrhagic shock. Conservative therapy with only thoracic drainage with close monitoring of outflow and hemodynamic parameters was performed. In front of hemodynamic instability, an emergency video-assisted thoracoscopic surgery was performed. An apical bulla adhering to the parietal pleura has been identified as the source of the bleeding. The resection of the bullae and electrocauterization of the bleeding adhesion were effectuated. The hemostasis was easily achieved. The actual experience suggests that video-assisted thoracoscopic surgery should be performed as soon as possible after the diagnosis of spontaneous hemopneumothorax. Indeed, conservative therapy with chest drainage should only be performed as bridge to recovery for the stabilization before the video-assisted thoracoscopic surgery.

## Introduction

Spontaneous hemopneumothorax (SHP) corresponds to an accumulation of blood and air in the pleural cavity outside of any traumatic context. The first description of SHP is credited to Laennec, who described it following a postmortem in 1829 [1]. This rare disorder is more common in men than in women and the gender difference is considerably larger than in spontaneous pneumothorax (SP) [2]. It affects approximately 0.5-11.6% of patients with SP [2,3]. It can be life-threatening situation due to rapid ventilatory collapse, hypovolemic shock and in front of misdiagnosed cause of unexplained hypovolemia. We report a case of poorly tolerated SHP. This condition has opposed a management and therapeutic challenge.

## Patient and observation

A 31-year-old male was admitted in the pulmonology department; after a short course in the emergency department; for a sudden onset of right-sided chest pain, shortness of breath, dry cough evolving since one day and worsened last 2 hours. He was tall and thin, chronic smoker with 10-years history of smoking with 15 cigarettes per day. He had no chirurgical, traumatic or medical past history and he experienced no other recent complaints especially hemoptysis or fever. He was working as a waiter in a restaurant and had no similar previous symptoms.

**Figure 1:**
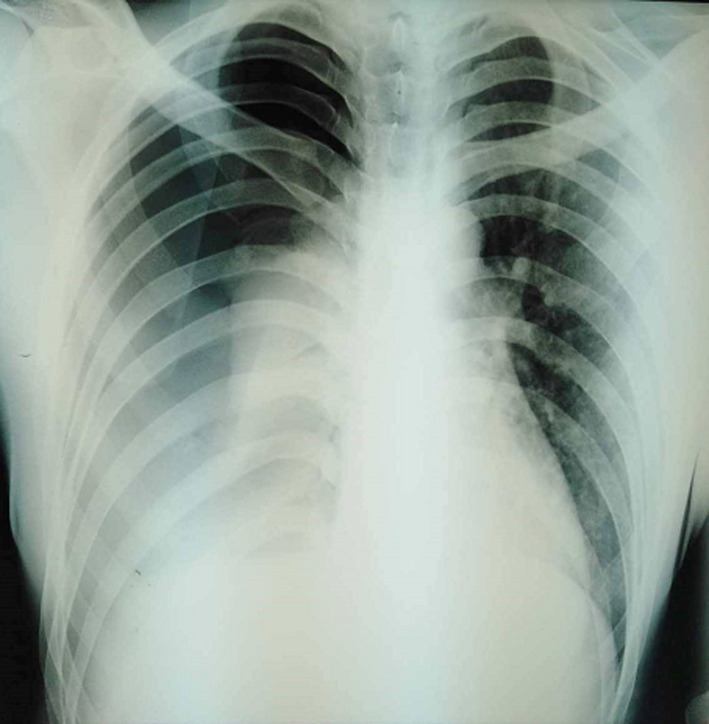
chest X-ray showing large, complete and compressive right-sided pneumothorax associated to pleural effusion

**Figure 2:**
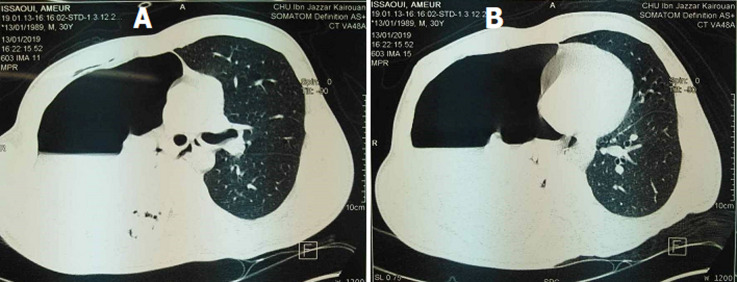
(A,B) thoracic computed tomography (CT) scan showing a large right-sided compressive hydropneumothorax with lung collapse and pleural effusion

On first examination by the emergency team, the patient was conscious. He was afebrile. He had blood pressure of 100/60mmHg and heart rate of 100beats/min. He had respiratory rate of 38 breaths/min with an oxygen saturation of 92% on room air. At physical examination, the patient was dyspneic with painful distress. Pulmonary examination was significant for abolished breathing sounds in the right hemithorax. Electrocardiography (ECG) showed normal sinus rhythm with tachycardia of 110beats/min and chest X-ray (CXR) evidenced a large, complete and compressive right-sided pneumothorax with pleural effusion (Figure 1). A thoracic computed tomography (CT) scan was performed showing a large right-sided hydropneumothorax with lung collapse (Figure 2). A 24-Fr thoracic drain was inserted in the medio-axillair line in the 5^th^ intercostal space under local anesthesia and in sterile conditions. Air and 900ml of blood were immediately evacuated and then the outflow of blood stopped.

**Figure 3:**
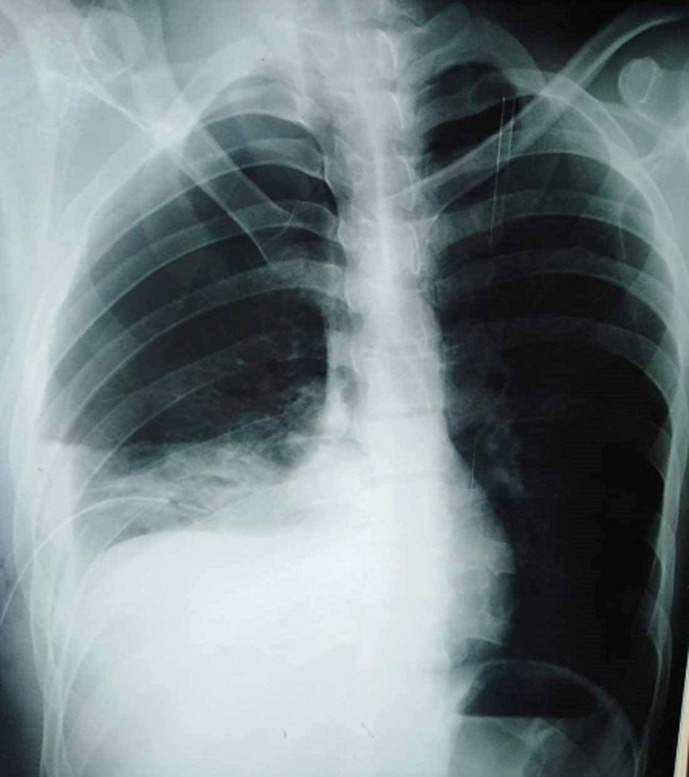
control chest X-ray showing recurrence and increasing of the right pleural effusion and chest drainage in place

A CT scan was then performed to evaluate the position of the thoracic drain, the re-expansion of the right lung and to identify the source of bleeding. It revealed a thoracic drain in the pleural cavity with adequate lung re-expansion, no contrast extravasation signs, no underlying mass or vascular malformation but a dystrophic bullous emphysema and a small blood collection in the right-sided pleural cavity. In front of the stabilization of vital signs and the interruption of bleeding, we decided to continue conservative therapy with chest drainage and not to proceed to surgical intervention. Twenty hours after the drainage, the patient developed obvious hemodynamic instability with blood pressure of 80/50 mmHg, heart rate of 135 beats/min, mottling and drop of hemoglobin level from 14.1 g/dl to 9.1 g/dl. Another 400 ml of blood flowed out from the chest tube. An urgent CXR revealed the increasing of the right pleural effusion (Figure 3).

**Figure 4:**
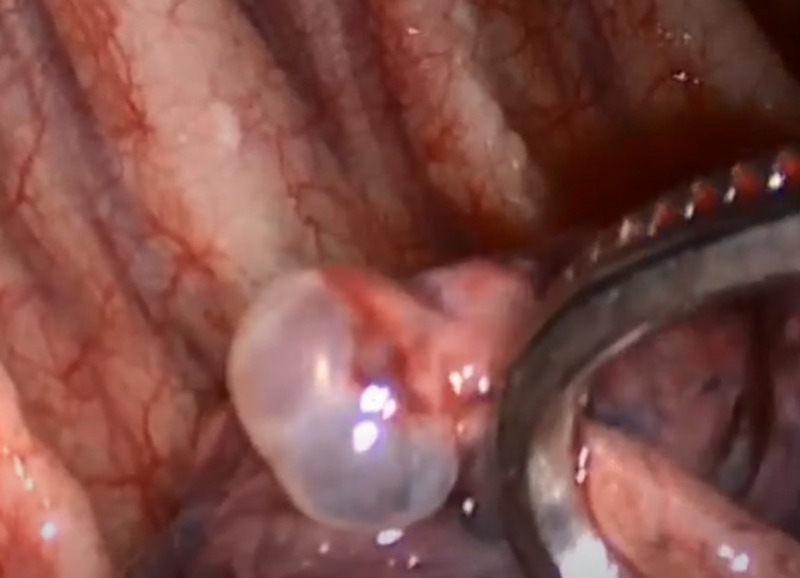
thoracoscopic visualization of a small apical bullae in the right upper lobe with a collection of blood in the pleural cavity

In front of the persistence of hemodynamic instability despite resuscitation and red blood cell transfusion, the medical team chose to indicate video-assisted thoracic surgery (VATS) to accomplish definitive hemostasis of the bleeding source. The patient was transferred to the cardiothoracic surgical ward and VATS was performed. Approximately 800 ml of blood and clots in the pleural cavity was removed. A small apical bulla was found in the right upper lobe near a pleural adhesion ruptured (Figure 4). The resection of the bullae, electrocauterization of the bleeding adhesion, irrigation of the pleural cavity and mechanical pleurodesis were effectuated. The hemostasis was easily achieved and two chest tubes were placed (anteroapical and posteroapical).

Two days later, the two chest tubes were removed in front of the non-recurrence of air and blood flow, the complete re-expansion of the right lung on CXR and the stable hemoglobin level of 12 g/dl after red blood cell transfusion. The patient was discharged 10 days after the procedure in front of clinical and radiological stability. Two weeks later, he was seen for routine check-up, no complaints were noticed and CXR confirmed full expansion of the lung.

## Discussion

SHP is defined as the accumulation of more than 400 ml of blood in the pleural cavity in association with primary spontaneous pneumothorax (PSP) without any traumatic or chirurgical context. Almost all described cases of SHP occurred as a complication of a PSP. The incidence of SHP has been reported to be around 1-12% of all PSP [4]. It is most common in young men in the age range of 20-40 years. Fry *et al*. reported that the incidence of SHP was found to be 25.4 times higher in male patients than in female patients and they suggested that this male predisposition may be due to the additional strength in exercise [5]. SHP is a well-documented disorder, however, it is rarely encountered in clinical practice. It can be life threatening due to the hemodynamic instability with hypovolemic shock, so a prompt diagnosis and therapeutic intervention are required.

While there are no specific guidelines for the management of patients diagnosed with SHP, the clinical features of SHP are dramatic and aggressive management is required. Therapeutic protocols are determined individually based on the patient´s clinical appearance and condition. The goals of therapeutic management include resuscitation, red blood cell transfusion, hemostasis and re-expansion of the lung. Recently, there seems to be a trend increasingly favorable towards early surgical intervention and the clinical outcomes in patients who undergo surgical intervention are better than those in patients managed with conservative therapy with chest drainage alone. With our patient, despite an initial improvement in clinical condition by chest drainage, the hemodynamic state worsened later and we failed to achieve hemostasis with conservative therapy. This finding confirms the hypothesis that surgical intervention should be performed as soon as possible after the diagnosis of SHP.

Hsu *et al*. reported that 87.6% out of 201 patients with SHP required surgical intervention after chest drainage. They advocated conservative therapy with chest drainage only in patients with stable hemodynamic state and those who have no continuous bleeding and no persistent air leaks. The indications for urgent surgical intervention include an aspiration of more than 1.5 liters of blood, hypovolemic shock, persistent air leak, clot empyema and pachypleuritis [6]. Chong *et al*. reported that 20-30% of patients that initially achieved hemostasis with conservative therapy, ultimately required surgical intervention or showed a prolonged hospital stay due to reactive fluid collection, persistent air leak or infectious complications [7]. Surgical strategies for the management of SHP include open thoracotomy or VATS.

Recently, early intervention of SHP by VATS is recommended, with better clinical outcomes. VATS has the advantages of less postoperative pain, improved pulmonary function and a decreased length of hospital stay then open thoracotomy. There are many successful experiences with VATS in the treatment of SHP currently reported. Yu *et al*. reported that patients who underwent early VATS had a shorter hospital length of stay, less bleeding and less frequently required transfusion, shorter period of drainage, than those who underwent conservative therapies [8]. VATS allows a better view of the pleural cavity, identifying and stopping the bleeding directly, evacuation of clotted blood from pleural cavity, sealing the area of air leak with endoscopic stapler and mechanical pleurodesis, as well as placement of the drainage tube under direct thoracoscopic vision all performed under minimal access trauma [9].

Hemostasis is required in surgery. Bleeding in SHP could be explained by different pathophysiological mechanisms. In our case, a ruptured adhesion between apical bullae and the parietal pleurae has been identified as the source of the bleeding and hemostasis was easily achieved after bullectomy and electrocauterization of the bleeding adhesion. The most common mechanism of bleeding is the tearing of aberrant vessels between the parietal pleura and adhesion bullae following pneumothorax-induced lung collapse [8,10]. The other mechanisms could be a torn adhesion between the parietal and visceral pleurae or a rupture of vascularized bullae or lung parenchyma. In 29.6-35% cases, the origin of bleeding cannot be identified.

## Conclusion

Although a rare entity, diagnosis of SHP must be considered in young men presenting with spontaneous onset of chest pain and dyspnea with radiograph findings of hydropneumothorax and/or signs of shock. The actual experience suggest that aggressive and immediate VATS should be performed as soon as possible after the diagnosis of SHP. Indeed, conservative therapy with chest drainage should only be performed as bridge to recovery for the stabilization before the VATS.
